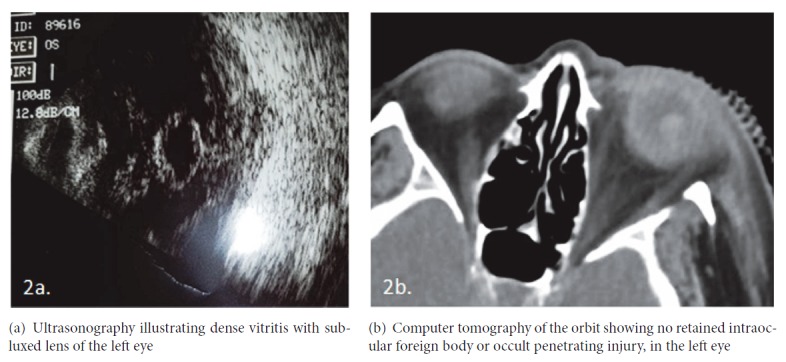# Endogenous* Clostridium perfringens* Panophthalmitis with Potential Entry Port from Diverticulitis Exacerbated by Proliferative Diabetic Retinopathy

**DOI:** 10.1155/2019/3462459

**Published:** 2019-03-04

**Authors:** Vamsee Neerukonda, Anny M. S. Cheng, Swetha Dhanireddy, Samuel Alpert, Han Y. Yin

**Affiliations:** ^1^SUNY Upstate Medical University, Department of Ophthalmology, Syracuse, NY, USA; ^2^Florida International University, Herbert Wertheim College of Medicine, Miami, FL, USA

## Abstract

**Purpose:**

To report a rapid endogenous fulminating panophthalmitis from* Clostridium perfringens* in a patient with diverticulitis and proliferative diabetic retinopathy.

**Methods:**

A 61-year-old female with poorly controlled diabetes mellitus, active proliferative diabetic retinopathy, and recent diverticulitis presented with conjunctival injection, ocular discharge, and sudden onset of painful vision loss of the left eye. Patient denied history of ocular trauma, intraocular surgery, or intravenous drug abuse. Examination revealed an erythematous, proptotic eye with restricted extraocular movements, mucopurulent discharge, diffuse corneal edema, and vitreous haze and cell. Orbital computed tomography (CT) confirmed no retained intraocular foreign body.

**Results:**

Despite 48 hours of treatment with systemic broad spectrum antimicrobial therapy (vancomycin, meropenem, and amphotericin B), patient underwent enucleation due to declined condition and progressive infection. Patient's culture revealed gram-positive bacillus microbes (*Clostridium perfringens*). Patient's subsequent CT abdomen showed resolved diverticulitis after antimicrobial therapy.

**Conclusion:**

Although rare,* Clostridium perfringens* infection can be a cause of rapid loss of vision from fulminate endogenous panophthalmitis. Urgent extensive systemic work-up to identify potential port of entry from visceral pathology and rapid removal of source of infection are pivotal to avoid high rate of mortality.

## 1. Introduction

*Clostridium perfringens* endophthalmitis is a rare but vision threatening infection. The source* Clostridium perfringens* is usually exogenous [1–6], and there is limited literature regarding an endogenous source. Endogenous* Clostridium perfringens* endophthalmitis can pose serious threats to both vision and is associated with high mortality [7, 8]. Previous reports demonstrated an underlying inflamed gallbladder [9], biliary infection [7], and acute abdomen [8] as causative sources. Herein, we report a case of endogenous* Clostridium perfringens* panophthalmitis in a patient with diverticulitis and proliferative diabetic retinopathy (PDR).

## 2. Case Report

**Figure 1:**
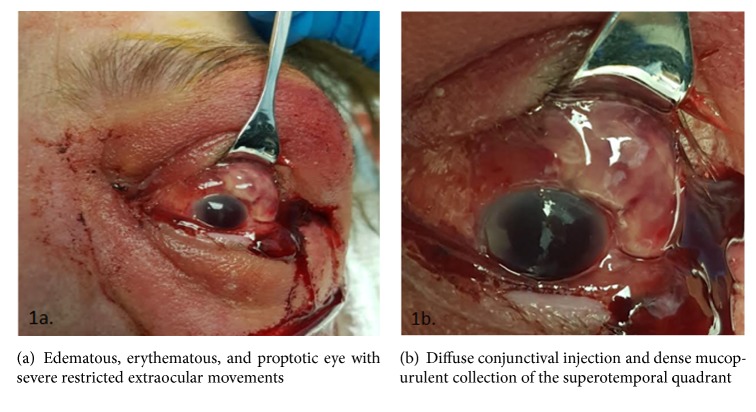
Panophthalmitis of the left eye.

A 61-year-old female with poorly controlled diabetes mellitus with severe bilateral PDR presented to the emergency department at Upstate University Medical Center. Initially, the patient presented to an outside emergency room with conjunctival injection, copious purulent discharge and associated painful vision loss to no light perception (NLP) in the left eye. Patient was then transferred to Upstate University Medical Center for higher level care and was evaluated by the ophthalmology service 18 hours after symptom onset. She underwent lateral canthotomy and cantholysis at the outside hospital 6 hours prior to arrival, due to severe pain and concern for orbital compartment syndrome; however patient endorsed complete loss of vision 12 hours prior in her left eye. She had no ocular trauma or history of intravenous drug abuse. However, she noted to have a two-month history of intermittent left abdominal pain and diarrhea, along with an abdominal computed tomography (CT) reporting diverticulitis. On presentation, examination revealed an edematous, erythematous, and proptotic left eye with severely restricted extraocular movements (Figure 1(a)). The anterior segment exam revealed diffuse conjunctival injection, mucopurulent discharge from superotemporal globe (Figure 1(b)), diffuse corneal edema and haze, and extensive fibrin in the anterior chamber. The dilated exam was limited due to corneal edema, diffuse anterior chamber reaction, and dense vitritis. Ophthalmic ultrasonography revealed a subluxed lens with diffuse vitritis (Figure 2(a)). Orbital CT confirmed no retained intraocular foreign body (Figure 2(b)) or occult penetrating injury.

Upon admission, patient's blood and specimens from ocular mucopurulent discharge were sent for gram stain and culture. The gram stain revealed gram-positive bacillus. The patient was admitted and started on intravenous (IV) broad spectrum antimicrobial therapy (vancomycin, meropenem, and amphotericin B). Due to progressive clinical decline with associated leukocytosis and encephalopathy 48 hours after initiating systemic antimicrobial treatment and gram stain results, she underwent enucleation of the left eye. Postoperatively, the patient completed a full course of IV vancomycin and meropenem.

Reflex anaerobic culture from the mucopurulent collection grew* Clostridium perfringens* and gross specimen from the enucleation revealed numerous gram-positive bacillus microbes. Anaerobic microbes seen on staining and samples were inoculated onto prereduced anaerobically sterilized Brucella blood agar, phenylethyl alcohol blood agar, kanamycin-vancomycin laked blood agar, and Bacteroides bile esculin agar (Oxyrase, Inc., Mansfield, OH). The inoculated plates were incubated at 35 degrees C anaerobically using the AnaeroPack-Anaero Anaerobic Gas Generator (Mitsubishi Gas Chemical America, New York, NY). After 48 hours of incubation, 2+ growth of a gram-positive bacillus was noted on the Brucella blood agar. These colonies were identified as* Clostridium perfringens* by Matrix-Assisted Laser Desorption Ionization-Time of Flight Mass Spectrometry (Vitek MS, bioMerieux, Inc., Durham, NC). No other growth was observed.

Amphotericin B was discontinued once fungal etiology was excluded. Subsequently, both the periorbital edema and erythema resolved. Additionally, the patient regained consciousness, as her encephalopathy and infection improved. She was subsequently transferred to local rehabilitation facility. A repeat CT abdomen demonstrated the previous inflammation had resolved, likely due to the aggressive inpatient broad spectrum antibiotics. No comorbid occult distal gastrointestinal malignancies were found.

## 3. Discussion

*Clostridium perfringens* is a rare ocular infection that results in devastating panophthalmitis with poor visual prognosis. The source of* Clostridium perfringens* is usually exogenous. The first report was described in a large series of 54 cases [1]. In that study, the authors noted that rapid painful visual loss developed within 12 hours; however all subjects had penetrating injuries. Since then, similar findings have been reported in isolated case reports after trauma [5], intravitreal injection [4], penetrating keratoplasty [3], cataract surgery [6], and among IV drug abusers [2]. Although the lateral canthotomy and cantholysis is a potential exogenous source of infection, this is much less likely in our patient, given the endogenous infection resulted in vision loss occurred 6 hours prior to the lateral canthotomy and cantholysis was performed.

There is limited literature regarding an endogenous source. To our knowledge, this is the fourth case of endogenous source of panophthalmitis resulting from* Clostridium perfringens*. The first case, described in 1974, showed a 68-year-old patient with endogenous endophthalmitis secondary to a biliary infection [7]. The gallbladder perforated despite surgical management and systemic IV antibiotics. The patient suffered associated rapid vision loss and the patient subsequently passed away from* Clostridium septicemia*. The second case, reported in 1992, was a patient with endogenous* Clostridium perfringens* endophthalmitis in setting of acute cholecystitis [9]. Despite surviving after surgical and IV interventions the patient's vision decreased to NLP vision within 24 hours of the globe perforating. The third case, reported in 2005, was acute painful loss of vision within 12 hours of developing an acute abdomen. The patient subsequently passed away from multiorgan failure from clostridial septicemia [8]. These 3 cases illustrate that endogenous* Clostridium perfringens* endophthalmitis can result in rapid complete loss of vision, and 2 out of 3 patients had fulminant systemic illnesses that resulted in mortality.

The clinical presentation of endogenous* Clostridium perfringens* endophthalmitis is a rare ocular manifestation. Although the portal of entry into the bloodstream in endogenous* Clostridium perfringens* infection remains unknown, studies have suggested that another Clostridium species,* Clostridium septicum*, has a strong association with diverticulosis, diabetes mellitus, severe arteriosclerotic disease, and occult visceral malignancy [10–12]. This patient had distal diverticulitis based on the abdominal CT two months prior to her ocular presentation. We propose that it is possible that the portal of entry into the bloodstream is from the distal sigmoid region. Our patient also had a history of poorly controlled diabetes with PDR changes affecting the inner blood retinal barrier. In addition to immunocompromised state in diabetes, PDR disrupts the inner blood retinal barrier by causing increased vascular permeability, loss of tight junctions [13], and a potential route for clostridial bacteremia to spread intraocularly. Further, given the immunocompromised state from diabetes, retinal neovascularization, and recent history of diverticulitis, our case highlights the importance of considering* Clostridium perfringens* as a cause of endogenous ocular dissemination. Further studies are needed to determine whether diverticulitis and PDR increase the risk of endogenous eye spreading in* Clostridium perfringens* infection.

In the setting of rapid painful loss of vision due to fulminate endophthalmitis, though it is rare,* Clostridium perfringens* must be considered as the endogenous infection may be associated with a life-threatening condition. Exogenous causes such as occult penetrating injury, intravenous drug use, and postsurgical procedures complications should be ruled out first. As this condition is rare, it is difficult to identify risk factors to alert an ophthalmologist to consider* Clostridium perfringens* as a possible etiology for orbital infection. Nevertheless, if endogenous endophthalmitis is suspected after excluding exogenous causes, a multidisciplinary systemic work-up with special emphasis on identifying the port of entry and promptly initiating appropriate broad spectrum antimicrobial coverage are warranted to avoid mortality.

## Figures and Tables

**Figure 2:**